# Prognostic analysis of curatively resected pancreatic cancer using harmonized positron emission tomography radiomic features

**DOI:** 10.1186/s41824-023-00163-8

**Published:** 2023-03-06

**Authors:** Masao Watanabe, Ryo Ashida, Chisato Miyakoshi, Shigeki Arizono, Tsuyoshi Suga, Shotaro Kanao, Koji Kitamura, Takahisa Ogawa, Reiichi Ishikura

**Affiliations:** 1 Department of Diagnostic Radiology, Kobe City Medical Center General Hospital, 2-1-1 Minatojima Minamimachi, Chuo-Ku, Kobe, 650-0047 Japan; 2 Department of Nuclear Medicine, University Clinic Essen, Hufelandstr. 55, 45147 Essen, Germany; 3 University of Duisburg-Essen and German Cancer Consortium (DKTK)-University Hospital, Hufelandstr. 55, 45147 Essen, Germany; 4 Department of Therapeutic Radiology, Kobe City Medical Center General Hospital, 2-1-1 Minatojima Minamimachi, Chuo-Ku, Kobe, 650-0047 Japan; 5 Department of Research Support, Center for Clinical Research and Innovation, Kobe City Medical Center General Hospital, 2-1-1 Minatojima Minamimachi, Chuo-Ku, Kobe, 650-0047 Japan; 6 Department of Surgery, Kobe City Medical Center General Hospital, 2-1-1 Minatojima Minamimachi, Chuo-Ku, Kobe, 650-0047 Japan; 7 Department of Orthopaedics and Spine Surgery, Graduate School, Tokyo Medical and Dental University, 1-5-45 Yushima, Bunkyo-Ku, Tokyo, 113-8519 Japan

**Keywords:** PET radiomics, Pancreatic cancer, Complete surgery, Harmonization, FDG PET/CT, Progression-free survival, Overall survival, Random forest

## Abstract

**Background:**

Texture features reflecting tumour heterogeneity enable us to investigate prognostic factors. The R package *ComBat* can harmonize the quantitative texture features among several positron emission tomography (PET) scanners. We aimed to identify prognostic factors among harmonized PET radiomic features and clinical information from pancreatic cancer patients who underwent curative surgery.

**Methods:**

Fifty-eight patients underwent preoperative enhanced dynamic computed tomography (CT) scanning and fluorodeoxyglucose PET/CT using four PET scanners. Using *LIFEx* software, we measured PET radiomic parameters including texture features with higher order and harmonized these PET parameters. For progression-free survival (PFS) and overall survival (OS), we evaluated clinical information, including age, TNM stage, and neural invasion, and the harmonized PET radiomic features based on univariate Cox proportional hazard regression. Next, we analysed the prognostic indices by multivariate Cox proportional hazard regression (1) by using either significant (*p* < 0.05) or borderline significant (*p* = 0.05–0.10) indices in the univariate analysis (first multivariate analysis) or (2) by using the selected features with random forest algorithms (second multivariate analysis). Finally, we checked these multivariate results by log-rank test.

**Results:**

Regarding the first multivariate analysis for PFS after univariate analysis, age was the significant prognostic factor (*p* = 0.020), and MTV and GLCM contrast were borderline significant (*p* = 0.051 and 0.075, respectively). Regarding the first multivariate analysis of OS, neural invasion, Shape sphericity and GLZLM LZLGE were significant (*p* = 0.019, 0.042 and 0.0076). In the second multivariate analysis, only MTV was significant (*p* = 0.046) for PFS, whereas GLZLM LZLGE was significant (*p* = 0.047), and Shape sphericity was borderline significant (*p* = 0.088) for OS. In the log-rank test, age, MTV and GLCM contrast were borderline significant for PFS (*p* = 0.08, 0.06 and 0.07, respectively), whereas neural invasion and Shape sphericity were significant (*p* = 0.03 and 0.04, respectively), and GLZLM LZLGE was borderline significant for OS (*p* = 0.08).

**Conclusions:**

Other than the clinical factors, MTV and GLCM contrast for PFS and Shape sphericity and GLZLM LZLGE for OS may be prognostic PET parameters. A prospective multicentre study with a larger sample size may be warranted.

## Background

Patients with pancreatic cancer have a low survival rate (Siegel et al. 2022). In 2022, pancreatic cancer was estimated to occur in 62,210 patients in the USA with an estimated 49,830 deaths (Siegel et al. 2022). In fewer than half of the cases, patients can undergo curative R0 complete surgery, which is essential for prolonged survival (Strobel et al. 2019). However, the recurrence rate even after curative surgery is relatively high. Thus, the classification of patients with curative surgery into groups with prolonged survival and short survival is essential in precision medicine for choosing the best treatment option. It might be possible to classify patients according to the recurrence probability to select close monitoring after surgery or the completion of adjuvant chemotherapy.

Fluorodeoxyglucose positron emission tomography/computed tomography (FDG PET/CT) is an essential imaging modality for diagnosing the tumour stage to estimate the probability of recurrence and to evaluate tumour remission after chemotherapy (Ha et al. 2017; Beukinga et al. 2017). In addition to conventional quantitative indices, including standardized uptake value (SUV), metabolic tumour volume (MTV), and total lesion glycolysis (TLG), textural features are valuable tools for assessing tumour heterogeneity and predicting patient outcomes (Ha et al. 2017; Beukinga et al. 2017).

However, textural features are sensitive to differences between PET scanners, PET algorithms, parameters of acquisition and reconstruction, as well as the voxel size, i.e. the “batch effect” (Orlhac et al. 2018; Goh et al. 2017). Mitigation of the batch effect is critical, mainly when multiple PET scanners are used (Orlhac et al. 2018). Orlhac et al. successfully mitigated the batch effect and normalized textural features with the R package *ComBat* using empirical Bayes methods, as well as the *LIFEx* freeware for radiomic feature calculation (Orlhac et al. 2018; Nioche et al. 2018; Johnson et al. 2007; Fortin et al. 2017, 2018). *ComBat* is robust even for small sample sizes (Johnson et al. 2007) on the condition that each group difference is not substantial and that the group design is not unbalanced (Goh et al. 2017; Nygaard et al. 2016). *ComBat* has been successfully applied in several studies of FDG PET/CT texture analysis (Dissaux et al. 2020; Nakajo et al. 2021).

In textural analyses of FDG PET/CT images, several studies demonstrated the prognostic utility of textural features of pancreatic cancer (Cui et al. 2016; Belli et al. 2018; Yoo et al. 2020; Kim and Kim 2021; Wei et al. 2021; Xing et al. 2021; Toyama et al. 2020). Among them, however, reports are rare that comprised only patients with pancreatic cancer who underwent complete curative surgery and used a harmonization method for radiomic features.

This study aimed to identify factors including harmonized PET radiomic features and clinical information significantly associated with the prognosis of pancreatic cancer patients who underwent curative surgery.

## Materials and methods

### Patients

**Fig. 1:**
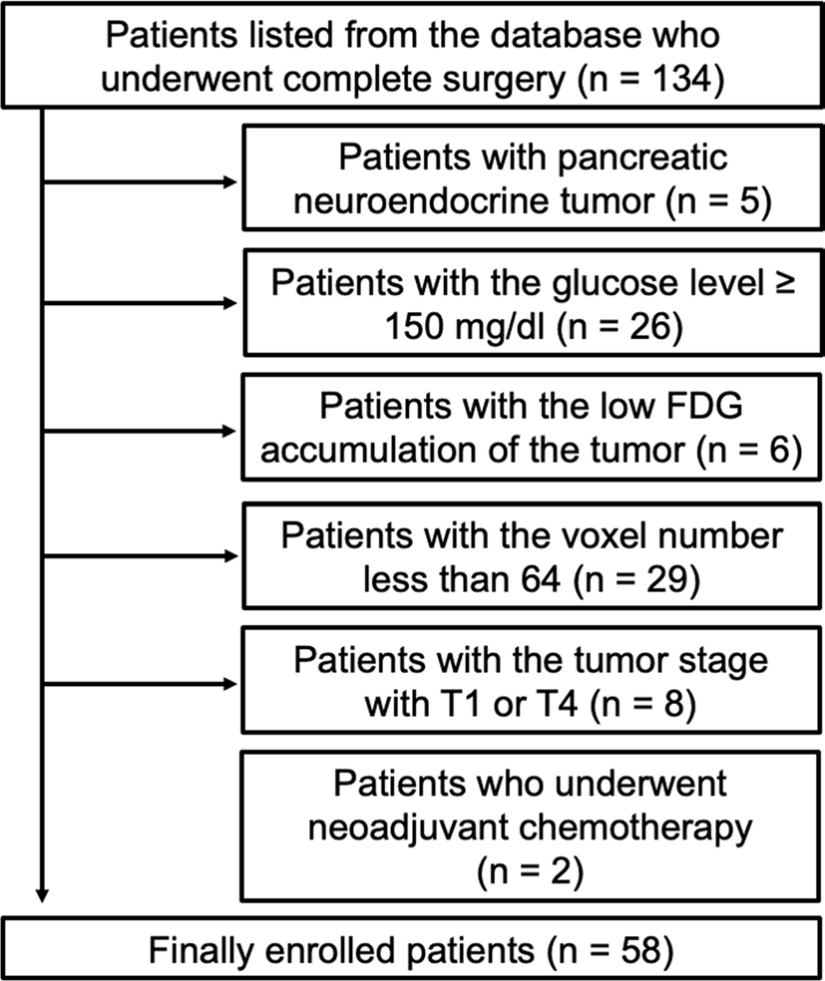
Schematic diagram of patient enrolment

We retrospectively searched the PET database of our institution and identified 134 consecutive patients who underwent pathologically complete curative surgery (R0) with sufficient clinical information and pathological assessment of the surgical specimen between July 2011 and March 2020. Among them, we excluded five patients with neuroendocrine tumours of the pancreas. Considering the guidelines of the European Association of Nuclear Medicine (EANM) 2015 (Boellaard et al. 2015), we also excluded 26 patients because their blood glucose level was equal to or greater than 150 mg/dl, although one of the main symptoms of patients with pancreatic cancer is hyperglycaemia. The FDG accumulation of the primary lesions in six patients was not high enough to secure a reproducible region of interest in the PET workstation. We also excluded 29 patients whose lesion voxel number in FDG PET/CT was less than 64 voxels, the threshold to extract textural features of FDG PET/CT (Kirienko et al. 2018; Orlhac et al. 2014). Then, we excluded eight patients with tumour (T) stage T1 (*n* = 7) or T4 (*n* = 1) to minimize covariance (Orlhac et al. 2022). Finally, we excluded two patients who underwent preoperative neoadjuvant chemotherapy. In total, we excluded 76 patients and enrolled 58 patients in our study (Fig. 1).

All enrolled patients (*n* = 58, 31 men and 27 women) preoperatively underwent dynamic contrast-enhanced CT and FDG PET/CT. The age of the enrolled patients was 69.2 ± 10.9 years (mean ± SD). The patient characteristics are summarized in Table 1. TNM staging was based on the Union for International Cancer Control (UICC) eighth edition. The pathological TNM staging of each patient was based on the surgical specimens. The differentiation of the adenocarcinomas (*n* = 55), i.e. from well-differentiated to poor-differentiated, was heterogeneous even in each primary pancreatic lesion; thus, the classification of these subtypes into one of three types of differentiation, i.e. well, moderate, and poor, was quite difficult. Among the 55 patients whose primary lesion histology was adenocarcinoma, there was one patient each with intraductal papillary mucinous adenocarcinoma, and adenocarcinoma with a small focal component of squamous differentiation.

**Table 1:** Patient characteristics (*n* = 58)

Age, mean ± SD (range), year	69.2 ± 10.9 (34–86)
Tumour location, *n*	
Head/body/tail	39/4/15
Pathological stage (UICC 8th edition), *n*	
T2/T3	42/16
N0/N1/N2	17/19/22
Stage IB/IIA/IIB/III	12/5/19/22
Histology, *n*	
Adenocarcinoma/adenosquamous/anaplastic	55/2/1
Surgery, *n*	
SSPPD/DP/TP	38/17/3
Neural invasion (pos/neg)	49/9
Adjuvant chemotherapy, *n*	
TS-1/GEM/incomplete	19/4/35

*DP* distal pancreatectomy, *GEM* gemcitabine, *S-1* TS-1 (Tegafur, Gimeracil, Oteracil Potassium), *SSPPD* subtotal stomach-preserving pancreaticoduodenectomy, *TP* total pancreatectomy, *UICC* Union for International Cancer Control

Concerning the patients with a past history of malignancies before surgery for pancreatic cancer (*n* = 19), chemotherapy or surgery for past malignancies was recorded in two, six, four, two, and four patients treated for lymphoma, breast cancer, malignancies of the digestive system (gastric cancer, gastrointestinal stromal tumour, and ampullary cancer), gynaecological cancers (uterine corpus cancer and uterine cervical cancer), and cancers of the urinary tract (renal cell carcinoma, urothelial cancer, and prostate cancer), respectively. The remaining patient had both gastric cancer and prostate cancer previously.

This retrospective study was approved by the institutional review board at Kobe City Medical Center General Hospital (registration number zn200707). The requirement for informed consent was waived, and the document that provided the patients with the opportunity to refuse to be enrolled in this study was uploaded onto our institute's website. This study was based on the Strengthening the Report of Observational Studies in Epidemiology (STROBE) guidelines for cohort studies.

### Preoperative enhanced CT and FDG PET/CT scanning

All patients underwent preoperative enhanced dynamic CT scanning from chest to pelvis to confirm that each tumour lesion could be completely surgically resected. Four PET scanners were used in our study; that is, Discovery IQ (*n* = 11; GE Healthcare, Waukesha, WI, USA), Discovery 600 (*n* = 26; GE Healthcare), Discovery ST (*n* = 12; GE Healthcare), and Gemini TF (*n* = 9, Philips Healthcare, Amsterdam, Netherlands). Among them, the primary scanner, Discovery 600, was used in accordance with our institutional protocol (Shimizu et al. 2016). The specifications of these scanners are described in Table 2.

**Table 2:** Specifications of the four PET scanners used in this study

	Discovery IQ	Discovery 600	Discovery ST	Gemini TF
Matrix size	192 × 192	192 × 192	128 × 128	144 × 144
Slice thickness	3.26 mm	3.27 mm	3.27 mm	4 mm
Pixel size	2.6 mm	2.6 mm	4.7 mm	4 mm
FOV	500 mm	500 mm	600 mm	576 mm
Reconstruction	QCHD	VPHDS	VPHD	BLOB-OS-TF
Subset/iteration	Not available^a^	16/3	21/2	33/3
Acquisition time (min/bed)	2–2.5 (2.5)^b^	1.5–3 (2.5)^b^	2 (2)^b^	2 (2)^b^
Postfiltering FWHM (mm)	Not available	3	4.29	Not available

*BLOB-OS-TF* blob ordered-subsets time-of-flight, *FOV* field of view, *FWHM* full width at half maximum, *PET* positron emission tomography, *QCHD* Q. Clear HD (a type of Bayesian penalized likelihood, GE Healthcare), *VPHD* VUE-point HD, *VPHDS* VPHD with point spread function

^a^The definition of the number of subsets and its iteration does not exist in Bayesian penalized likelihood reconstruction because of its regularization algorithm

^b^The median acquisition time of the scanner is described in parentheses

All patients fasted for at least 4 h. The injection dose of FDG in all patients was 225.8 ± 62.0 MBq (mean ± SD). The duration from injection to FDG PET/CT scan was 57.2 ± 4.1 min (mean ± SD). The mean interval of the two preoperative scans between the enhanced dynamic CT scan and FDG PET/CT scan was 11.4 days. The blood glucose levels of all enrolled patients were less than 150 mg/dl in accordance with the 2015 EANM guidelines (Boellaard et al. 2015) and with the results of a previous study that investigated the influence of glucose levels on the FDG accumulation of tumours (Eskian et al. 2019).

### Preoperative, surgical, and postoperative treatment

Thirty-eight patients with tumours in the pancreatic head underwent subtotal stomach-preserving pancreaticoduodenectomy. Seventeen patients with tumours in the pancreatic body (*n* = 3) or pancreatic tail (*n* = 14) underwent distal pancreatectomy. One patient who underwent distal pancreatectomy also underwent curative partial gastrectomy at the same time because of concurrent gastric cancer. The remaining three patients (pancreatic head, *n* = 1; pancreatic body, *n* = 1; and pancreatic tail, *n* = 1) underwent total pancreatectomy (TP). One patient with pancreatic tail cancer underwent TP because he had received subtotal stomach-preserving pancreaticoduodenectomy for ampullary cancer of the duodenum 4 years before TP. Another reason for TP was a pathologically positive margin of the on-site surgical specimen. In terms of neural invasion of primary pancreatic lesions, there were 49 positive and 9 negative patients.

Regarding postoperative chemotherapy, 19 patients completed four cycles of adjuvant S-1 therapy, and 4 patients completed approximately six cycles of adjuvant gemcitabine therapy. The remaining 35 patients failed to complete adjuvant chemotherapy treatment because of health conditions and adverse effects. Among the four PET scanners, there were no significant differences using chi-square test in terms of the patient characteristics listed in Table 1.

### Follow-up survey using imaging modalities

After completing surgery, all patients underwent postoperative surveys using laboratory data for carcinoembryonic antigen (CEA) and cancer antigen (CA)19-9 and imaging modalities, which mainly included enhanced CT scans, additional magnetic resonance imaging (MRI), and FDG PET/CT scans. In almost all cases, the postoperative laboratory survey was performed with intervals of 1–3 months which was, due to the retrospective nature of our study, partially irregular according to the patient’s health condition, and the postoperative imaging survey by CT scan was performed with an interval of approximately 3 months. Primarily, when the enhanced CT or laboratory data indicated recurrent lesions without certainty, the patients additionally underwent gadolinium-ethoxybenzyl diethylenetriamine pentaacetic acid (Gd-EOB-DTPA; Bayer, Leverkusen, Germany) MRI scans (*n* = 1) or FDG PET/CT scans (*n* = 6) to detect the recurrent lesion with confidence. All relapses of the enrolled patients were determined only by these imaging modalities because they are readily available at our institute.

For this retrospective study, a board-certified radiologist and nuclear medicine physician (M.W.; 13 years of experience) and an expert abdominal radiologist (S.A.; 21 years of experience) thoroughly checked all images (CT, MRI, and FDG PET/CT) again and finally determined the recurrence date.

### Data analysis including textural feature extraction

We used *LIFEx* version 6.30 (Nioche et al. 2018) to extract quantitative values, including SUVmax, SUVmean, MTV, TLG, and textural features. When determining the volume of interest of a primary pancreatic lesion, many primary pancreatic tumours did not have significant and sharply delineated FDG accumulation. Furthermore, the lesions were adjacent to pancreatic and duodenal vessels and the duodenal tract. To ensure the reproducibility of the volume of interest by semi-automatic segmentation (Belli et al. 2018), we applied Nestle’s method (*β* = 0.3), which is available in the *LIFEx* software (Orlhac et al. 2014; Maisonobe et al. 2013; Ha et al. 2019).

In addition to the conventional PET parameters (SUVmax, SUVmean, MTV, and TLG), sphericity, compacity, and other first-order histogram texture features, we extracted 31 textural features with higher order, including grey-level co-occurrence matrices (GLCM), grey-level run length matrix (GLRLM), neighbouring grey-level different matrix (NGLDM), and grey-level zone length matrix (GLZLM; Table 3).

**Table 3:** List of radiomic features extracted from FDG PET/CT

Matrix	Index
*First-order features*	SUVmax
	SUVmean
	Metabolic tumour volume (MTV)
	Total lesion glycolysis (TLG)
	Shape sphericity
	Shape compacity
	Discretized Histo Skewness
	Discretized Histo Kurtosis
	Discretized Histo Entropy Log10
	Discretized Histo Energy
*Second-order features*	
Grey-level co-occurrence matrix (GLCM)	Homogeneity
	Energy
	Contrast
	Correlation
	Entropy Log10
	Dissimilarity
*Higher-order features*	
Grey-level run length matrix (GLRLM)	Short-run emphasis (SRE)
	Long-run emphasis (LRE)
	Low grey-level run emphasis (LGRE)
	High grey-level run emphasis (HGRE)
	Short-run low grey-level emphasis (SRLGE)
	Short-run high grey-level emphasis (SRHGE)
	Long-run low grey-level emphasis (LRLGE)
	Long-run high grey-level emphasis (LRHGE)
	Grey-level nonuniformity for run (GLNU)
	Run length nonuniformity (RLNU)
	Run percentage (RP)
Neighbourhood grey-level different matrix (NGLDM)	Coarseness
	Contrast
	Busyness
Grey-level zone length matrix (GLZLM)	Short-zone emphasis (SZE)
	Long-zone emphasis (LZE)
	Low grey-level zone emphasis (LGZE)
	High grey-level zone emphasis (HGZE)
	Short-zone low grey-level emphasis (SZLGE)
	Short-zone high grey-level emphasis (SZHGE)
	Long-zone low grey-level emphasis (LZLGE)
	Long-zone high grey-level emphasis (LZHGE)
	Grey-level nonuniformity for zone (GLNU)
	Zone length nonuniformity (ZLNU)
	Zone percentage (ZP)

*FDG PET/CT* fluorodeoxyglucose positron emission tomography/computed tomography, *SUV* standardized uptake value

The main settings of the textural analysis were as follows: number of bins, 64; bin size, 0.3; and minimum and maximum bounds of the resampling, 0–20 SUV. The voxel size was resampled to 3.0 × 3.0 × 3.0 mm (Nakajo et al. 2021; Reuzé et al. 2018). We referred to the reporting guidelines of the Image Biomarker Standardization Initiative (ISBI, version v11, revised on 17 Dec 2019) (Zwanenburg et al. 2016). Regarding the harmonization using *ComBat* (neuroCombat_Rpackage version 1.0.11, https://github.com/Jfortin1/ComBatHarmonization) (Johnson et al. 2007; Fortin et al. 2017, 2018), we performed the free online application for harmonization (https://forlhac.shinyapps.io/Shiny_ComBat/) (Orlhac et al. 2022, 2021). Before the harmonization among four PET scanners (batches), we confirmed that there were no significant differences in terms of MTV, TLG, Shape sphericity and Shape compacity among these four groups of patients using the Kruskal–Wallis test. This is because these PET features depend mainly on the definition of the volume of interest and not on the intensity of FDG accumulation in the primary tumour, which is little impacted by the batch effect.

### Statistical analysis

We used R Statistical Software version 4.1.3 for the analysis (Foundation for Statistical Computing, Vienna, Austria). Progression-free survival (PFS) was defined as the duration from the date of surgery to the date of relapse of pancreatic cancer. Overall survival (OS) was defined as the duration from the date of surgery to the date of death, in which living patients were censored at the last follow-up date. Optimal cut-off values of PET parameters were determined with Classification and Regression Tree (CART) analysis using the R package *rpart* (version 4.1.16) with a PFS cut-off of 300 days and with OS cut-off of 1275 days (42 months) (Strobel et al. 2019; Groot et al. 2018). We classified patients also according to their age (age > 70 vs. age ≤ 70), tumour location (head vs. body-tail), T stage (T2 vs. T3), N stage (N0 vs. N1-3), and presence of neural invasion (positive vs. negative) into two groups.

For univariate analysis using Cox proportional hazard regression model for PFS and OS, we evaluated the clinical information in terms of age, sex, T stage, N stage, tumour location, presence of neural invasion, as well as the PET quantitative radiomic parameters stated above, by referring to the previous literature (Yamamoto et al. 2015). We did not evaluate CA19-9 in this study because CA19-9 can be elevated in patients with biliary obstruction, which can be caused by pancreatic cancer irrespective of tumour progression (Mizrahi et al. 2020; Hidalgo 2010).

The significant and borderline significant prognostic indices in the univariate analysis entered the multivariate analysis using Cox proportional hazard regression model for PFS and OS (first multivariate analysis) (Nakajo et al. 2021; Antunovic et al. 2019). Considering a large number of prognostic indices for the statistical analysis, we determined the rank of Gini importance calculated from the decrease in Gini impurity for feature selection by using the algorithm of random forest with tenfold cross-validation provided by the R package *caret* (version 6.0–93) by referring to the previous literature (Toyama et al. 2020; Menze et al. 2009). Then, by checking the graph of the rank regarding Gini importance of prognostic factors (clinical indices and PET parameters) exported from the R package as in the previous literature (Toyama et al. 2020), the high-rank prognostic indices were evaluated by another multivariate analysis for PFS and OS (second multivariate analysis) to confirm the results (Nakajo et al. 2022). The significant and borderline significant prognostic indices in either the first or second multivariate analysis were confirmed using Kaplan–Meier survival curves and log-rank tests. Indices with *p* values < 0.05 and with *p *values ≥ 0.05 but < 0.1 were considered statistically significant and borderline significant, respectively.

## Results

All prognostic statuses of the enrolled patients were followed up until the end of July 2021. The mean follow-up period from surgery was 29.2 months. Regarding PFS, 37 (63.8%) patients relapsed. Of all the patients, 21 (36.2%) patients survived, 25 (43.1%) patients died, and 12 (20.7%) patients were lost to follow-up. Among 19 patients who had other previous malignancies, 16 patients were without recurrence, 2 patients had stable disease, and the remaining patient had progressive disease at the end of the follow-up. All 19 patients did not die of these previous malignancies.

The results of the univariate and the first multivariate Cox proportional hazard regression analyses for PFS and OS are summarized in Tables 4 and 5. For the univariate analysis for PFS, NGLDM busyness and GLZLM LZLGE were significant prognostic factors (*p* = 0.036 and 0.019, respectively), and age, MTV, GLCM contrast and GLZLM LZE were borderline significant prognostic factors (*p* = 0.080, 0.072, 0.076 and 0.055, respectively). For the univariate analysis for OS, neural invasion, SUVmax, Shape sphericity, Histogram kurtosis, GLCM contrast, GLCM dissimilarity, GLZLM HGZE, GLZLM SZHGE were significant prognostic factors (*p* = 0.043, 0.047, 0.049, 0.034, 0.048, 0.048, 0.043 and 0.043, respectively), and TLG, GLCM homogeneity, GLRLM HGRE, GLRLM SRHGE, GLRLM GLNU, GLZLM LZLGE and GLZLM GLNU were borderline significant prognostic factors (*p* = 0.095, 0.078, 0.076, 0.076, 0.082, 0.085 and 0.054). Other clinical indices and PET radiomic features were not significant or borderline significant prognostic indices in the univariate analysis. Regarding the first multivariate analysis of PFS using the results of univariate analysis, age was the significant prognostic factor (*p* = 0.020), and MTV and GLCM contrast were borderline significant prognostic factors (*p* = 0.051 and 0.075, respectively). Regarding the first multivariate analysis of OS using the results of univariate analysis, neural invasion, Shape sphericity and GLZLM LZLGE were significant prognostic factors (*p* = 0.019, 0.042 and 0.0076).

**Table 4:** Univariate and multivariate analyses of progression-free survival

	Univariate analysis		Multivariate analysis	
	Hazard ratio (95% CI)	*p *value	Hazard ratio (95% CI)	*p *value
Age	0.93–3.59	0.080	1.15–4.74	0.020
MTV	0.10–1.10	0.072	0.08–1.01	0.051
GLCM contrast	0.25–1.07	0.076	0.18–1.09	0.075
NGLDM busyness	0.18–0.94	0.036	0.17–3.93	0.81
GLZLM LZE	0.25–1.01	0.055	0.26–3.01	0.85
GLZLM LZLGE	0.19–0.86	0.019	0.14–4.14	0.75

*CI* confidence interval, *MTV* metabolic tumour volume, *GLCM* grey-level co-occurrence matrix, *NGLDM* neighbourhood grey-level different matrix, *GLZLM* grey-level zone length matrix, *LZE* long-zone emphasis, *LZLGE* long-zone low grey-level emphasis

**Table 5:** Univariate and multivariate analyses of overall survival

	Univariate analysis		Multivariate analysis	
	Hazard ratio (95% CI)	*p* value	Hazard ratio (95% CI)	*p* value
Neural invasion	0.05–0.95	0.043	0.03–0.73	0.019
SUVmax	0.08–0.98	0.047	0.08–7.06	0.81
TLG	0.23–1.13	0.095	0.15–3.11	0.63
Shape sphericity	0.09–0.99	0.049	0.03–0.94	0.042
Histogram kurtosis	0.12–0.92	0.034	0.08–3.54	0.50
GLCM contrast	0.16–0.99	0.048	0.01–5.93	0.35
GLCM homogeneity	0.16–1.10	0.078	0.07–21.16	0.89
GLCM dissimilarity	0.16–0.99	0.048	NA*	NA*
GLRLM HGRE	0.15–1.10	0.076	0.05–79.60	0.74
GLRLM SRHGE	0.15–1.10	0.076	NA*	NA*
GLRLM GLNU	0.22–1.10	0.082	0.62–7.51	0.23
GLZLM HGZE	0.12–0.97	0.043	0.02–30.49	0.91
GLZLM SZHGE	0.12–0.97	0.043	NA*	NA*
GLZLM LZLGE	0.23–1.10	0.085	0.05–0.63	0.0076
GLZLM GLNU	0.20–1.01	0.054	0.09–2.07	0.29

*CI* confidence interval, *SUVmax* maximum standardized uptake value, *TLG* total lesion glycolysis, *GLCM* grey-level co-occurrence matrix, *GLRLM* grey-level run length matrix, *HGRE* high grey-level run emphasis, *SRHGE* short-run high grey-level emphasis, *(GLRLM) GLNU* grey-level nonuniformity for run, *LZLGE* long-zone low grey-level emphasis, *GLZLM* grey-level zone length matrix, *HGZE* high grey-level zone emphasis, *SZHGE* short-zone high grey-level emphasis, *LZLGE* long-zone low grey-level emphasis, *(GLZLM) GLNU* grey-level nonuniformity for zone, NA* not available due to multicollinearity between (1) GLCM contrast and GLCM dissimilarity (Pearson correlation coefficient *r* = 0.95 in pre-harmonization and 0.97 in post-harmonization), (2) GLRLM HGRE and GLRLM SRHGE (*r* = 0.9996 in pre-harmonization and 0.9993 in post-harmonization), and (3) GLZLM HGZE and GLZLM SZHGE (*r* = 0.99 in pre-harmonization and 0.99 in post-harmonization)

**Fig. 2:**
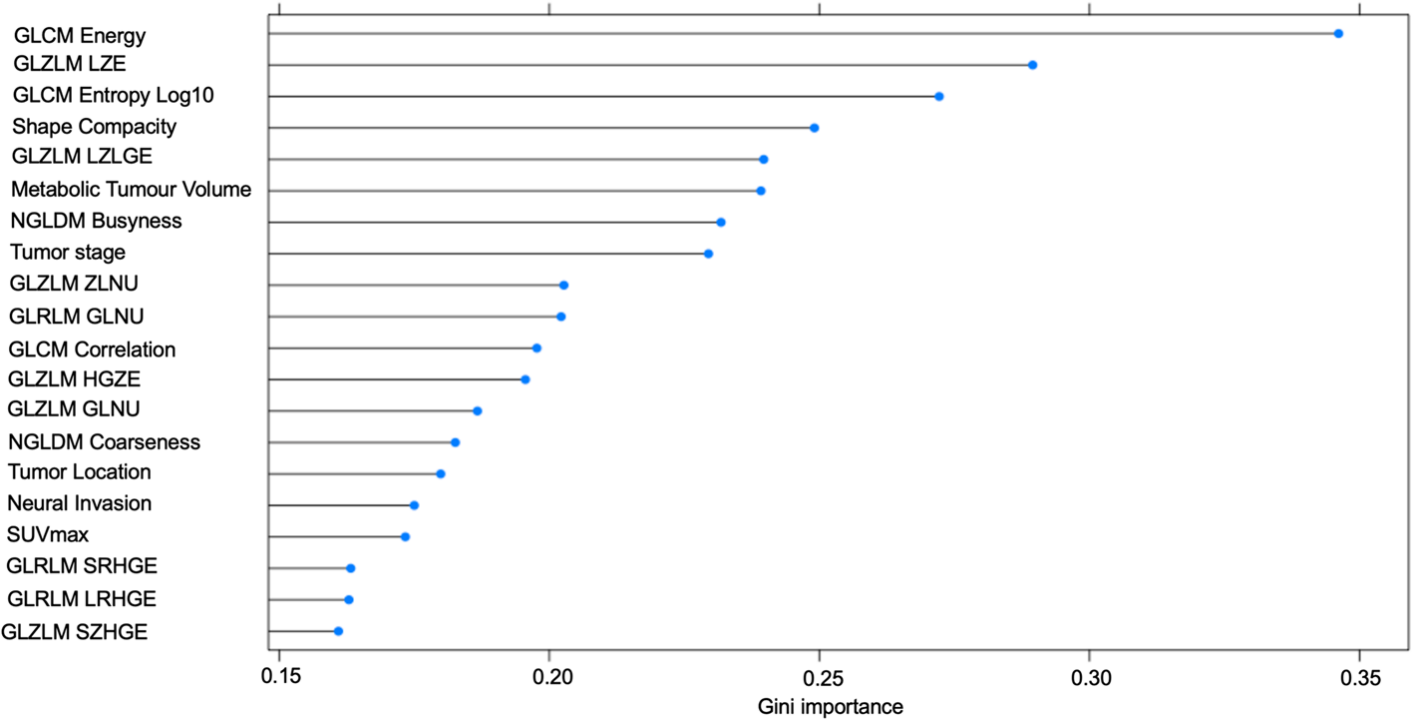
High-ranking clinical and radiomic features for PFS in terms of Gini importance. *PFS* progression-free survival

**Fig. 3:**
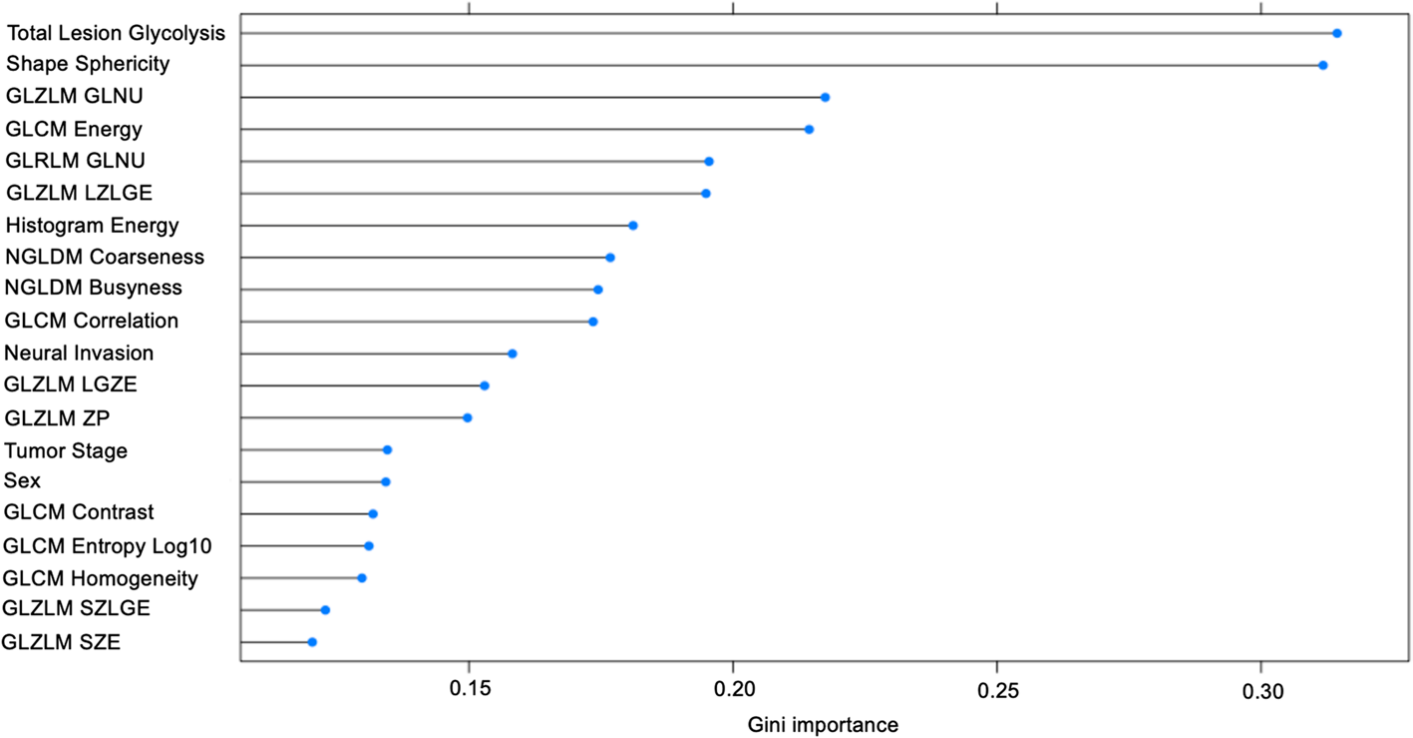
High-ranking clinical and radiomic features for OS in terms of Gini importance. *OS* overall survival

Concerning that there are a large number of clinical indices and PET radiomic features, we also evaluated Gini importance calculated from the decrease in Gini impurity for PFS and OS using the algorithm of random forest with tenfold cross-validation (Figs. 2, 3). We performed the second multivariate analysis for PFS and OS using the top six indices in Figs. 2 and 3. In the second multivariate analysis for PFS, only MTV was significant (*p* = 0.046) (Table 6). For OS, GLZLM LZLGE was significant (*p* = 0.047), and Shape sphericity was borderline significant (*p* = 0.088) (Table 7).

**Table 6:** Multivariate analyses of progression-free survival using random forest algorithms

	Hazard ratio (95% CI)	*p *value
MTV	0.08–0.98	0.046
Shape compacity	0.18–1.20	0.11
GLCM energy	0.00–Inf*	0.997
GLCM entropy Log10	0.00–Inf*	0.997
GLZLM LZE	0.24–2.93	0.78
GLZLM LZLGE	0.13–1.99	0.34

*CI* confidence interval, *MTV* metabolic tumour volume, *GLCM* grey-level co-occurrence matrix, *GLZLM* grey-level zone length matrix, Inf* with no upper limit

**Table 7:** Multivariate analyses of overall survival using random forest algorithms

	Hazard ratio (95% CI)	*p* value
TLG	0.22–3.18	0.80
Shape sphericity	0.08–1.19	0.088
GLCM energy	0.11–1.56	0.19
GLRLM GLNU	0.44–5.17	0.51
GLZLM LZLGE	0.15–0.99	0.047
GLZLM GLNU	0.13–2.09	0.37

*CI* confidence interval, *TLG* total lesion glycolysis, *GLCM* grey-level co-occurrence matrix, *GLRLM GLNU* grey-level run length matrix grey-level nonuniformity for run, *GLZLM* grey-level zone length matrix, *LZLGE* long-zone low grey-level emphasis, *(GLZLM) GLNU* grey-level nonuniformity for zone

**Fig. 4:**
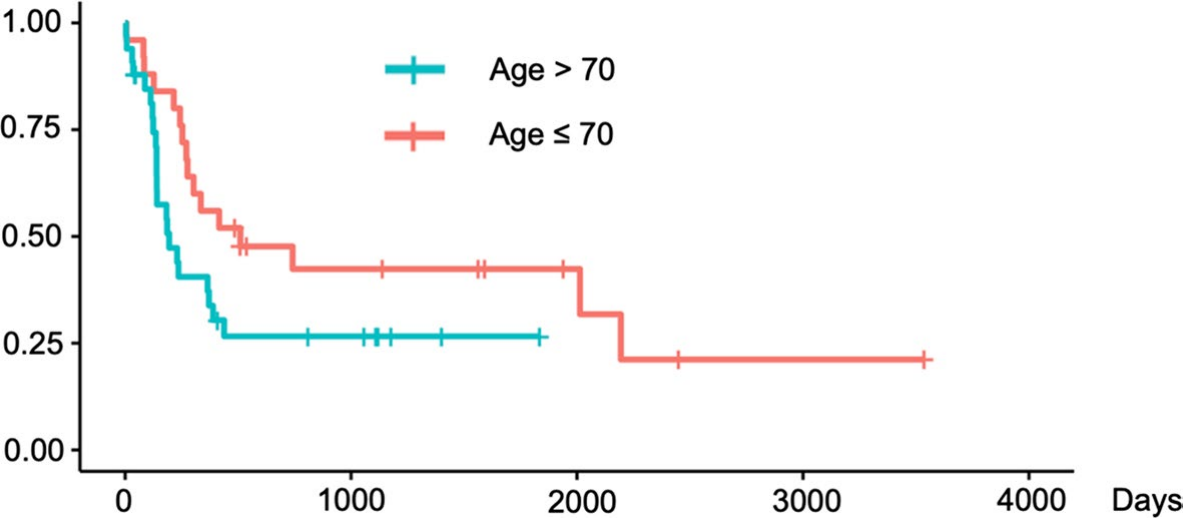
Kaplan–Meier survival curve analysis for PFS in terms of age. In the log-rank test, *p* value was 0.080. *PFS* progression-free survival

**Fig. 5:**
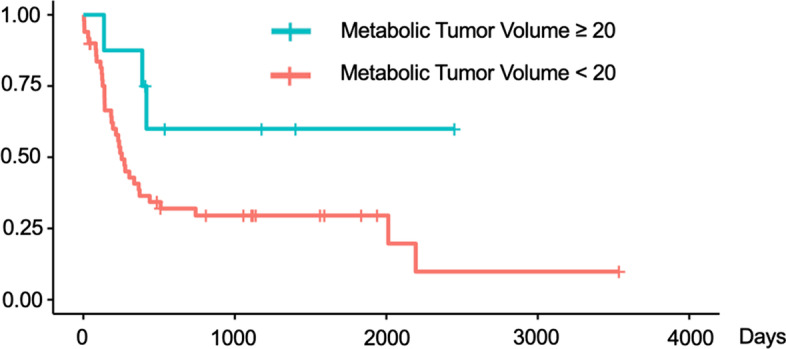
Kaplan–Meier survival curve analysis for PFS in terms of MTV. In the log-rank test, *p* value was 0.060. *PFS* progression-free survival, *MTV* metabolic tumour volume

**Fig. 6:**
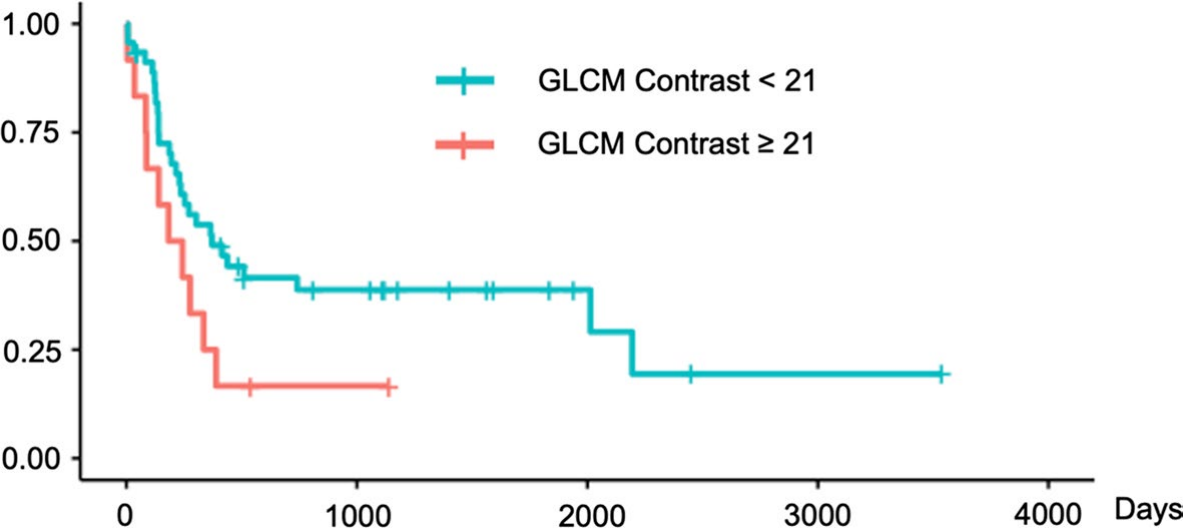
Kaplan–Meier survival curve analysis for PFS in terms of GLCM Contrast. In the log-rank test, *p* value was 0.070. *PFS* progression-free survival, *GLCM* grey-level co-occurrence matrix

**Fig. 7:**
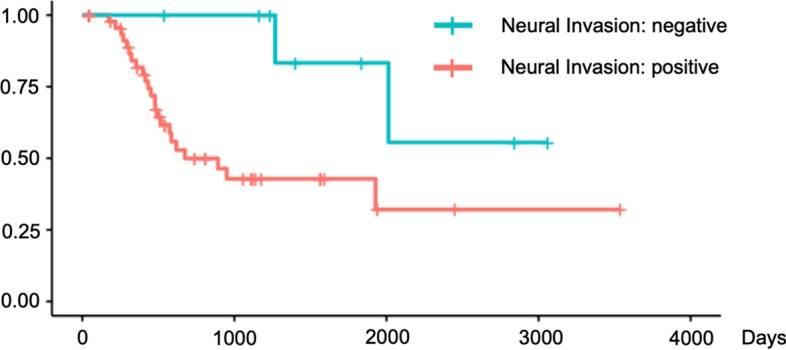
Kaplan–Meier survival curve analysis for OS in terms of neural invasion. In the log-rank test, *p* value was 0.030. *OS* overall survival

**Fig. 8:**
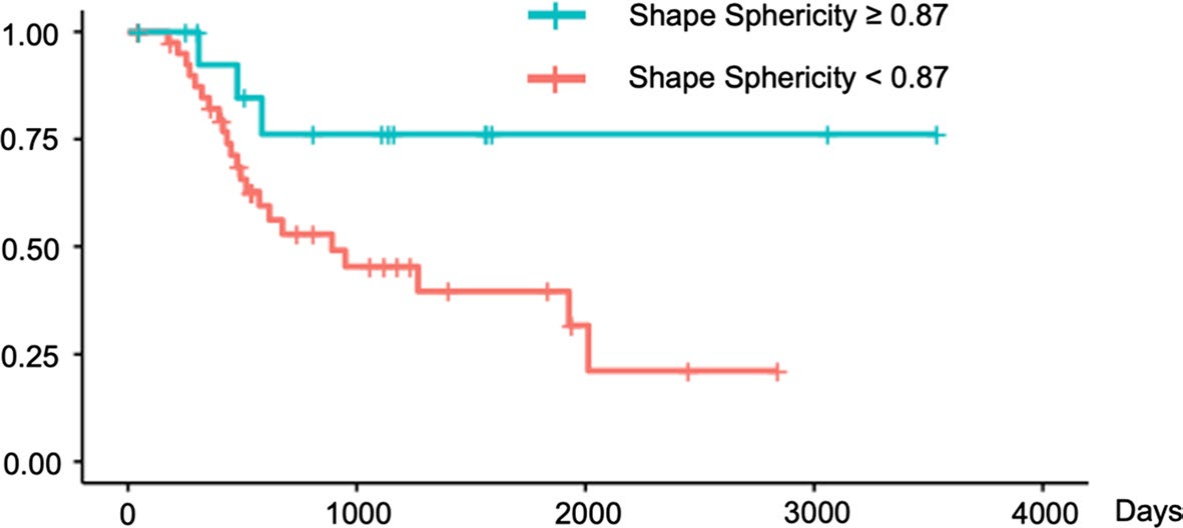
Kaplan–Meier survival curve analysis for OS in terms of Shape sphericity. In the log-rank test, *p* value was 0.040. *OS* overall survival

**Fig. 9:**
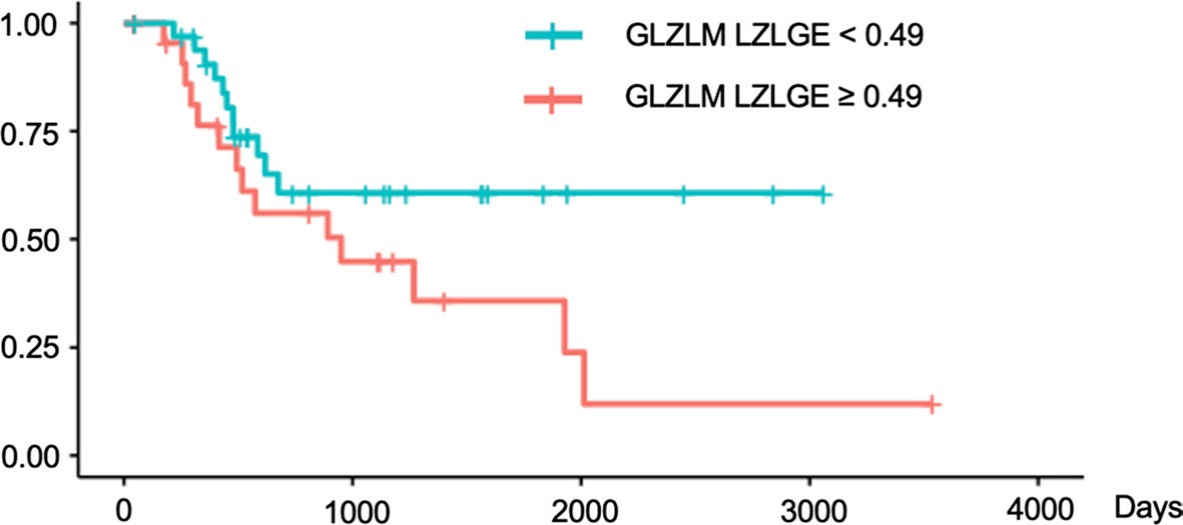
Kaplan–Meier survival curve analysis for OS in terms of GLZLM LZLGE. In the log-rank test, *p* value was 0.080. *OS* overall survival, *GLZLM* grey-level zone length matrix, *LZLGE* long-zone low grey-level emphasis

We validated these findings using Kaplan–Meier survival curves with log-rank tests for PFS (Figs. 4, 5, 6) and for OS (Figs. 7, 8, 9). In the log-rank test for PFS, age, MTV and GLCM contrast were borderline significant (*p* = 0.08, 0.06 and 0.07, respectively). In the log-rank test for OS, neural invasion and Shape sphericity were significant (*p* = 0.03 and 0.04, respectively), and GLZLM LZLGE was borderline significant (*p* = 0.08).

## Discussion

We investigated the clinical utility of PET radiomic features in patients with pancreatic cancer. For PFS, age was a significant prognostic factor, and MTV and GLCM contrast were borderline significant prognostic factors in the first multivariate analysis, whereas only MTV was significant in the second multivariate analysis. For OS, neural invasion, Shape sphericity and GLZLM LZLGE were significant in the first multivariate analysis, whereas GLZLM LZLGE was significant, and Shape sphericity was borderline significant in the second multivariate analysis. GLCM dissimilarity, GLRLM SRHGE and GLZLM SZHGE were not available in the first multivariate analysis for OS due to the multicollinearity between (1) GLCM contrast and GLCM dissimilarity, (2) GLRLM HGRE and GLRLM SRHGE, and (3) GLZLM HGZE and GLZLM SZHGE.

For feature selection of the prognostic indices for the multivariate analysis, we used the rank of Gini importance calculated from the decrease in Gini impurity by using random forest with tenfold cross-validation other than using results of the univariate analysis in accordance with the previous literature (Toyama et al. 2020; Menze et al. 2009). Random forest is an ensemble learner based on randomized decision trees, and the Gini impurity is a computationally efficient approximation to the entropy (Menze et al. 2009). The decrease in Gini impurity indicates how much the overall discriminative value of each feature contributed to the classification task (Menze et al. 2009). Random forest can be more useful and robust for feature selection than univariate analysis when the features are highly correlated with each other (Menze et al. 2009). In our PET radiomic study, PET radiomic features are classified into several texture categories, i.e. GLCM, GLRLM, NGLDM, and GLZLM, and there are a large number of PET features to be analysed for the prognostic statistics. In consideration of the α errors caused by the multiple univariate analyses, we performed a second multivariate analysis using the features selected by random forest algorithms.

For harmonization of the PET radiomic features among several PET scanners using *ComBat*, Johnson et al. proposed a method that robustly adjusts even batches with small sample sizes (Johnson et al. 2007). The utility of this harmonization algorithm in the FDG texture analysis was demonstrated in several studies (Orlhac et al. 2018; Dissaux et al. 2020; Nakajo et al. 2021). Interestingly, Nygaard et al. claimed that harmonization will reduce group statistical power, especially when the design of each group is unbalanced (Goh et al. 2017; Nygaard et al. 2016; Da-Ano et al. 2020). Dissaux et al. used *ComBat* for PET image harmonization, in which the numbers of lung cancer patients in batches of different PET scanner types were 27, 29, and 8 (Dissaux et al. 2020). Therefore, the patient number in each batch of our study (26, 12, 11, and 9 patients per PET scanner type) seemed to be acceptable. However, Orlhac et al. demonstrated it is desirable to secure a patient number of more than 20 in each batch for the harmonization using *ComBat* considering the variability of PET parameters (Orlhac et al. 2022). Although we enrolled only patients with tumour stages T2 (*n* = 42) or T3 (*n* = 16) to reduce the effect of this covariate (Orlhac et al. 2022), the number of patients in each batch might not have been enough in our study for the PET harmonization in a study of pancreatic cancer.

In PET radiomic studies, Toyama et al. and Kim et al. demonstrated that a TLG > 34.6 and a TLG > 63.95, respectively, allowed the prognostic stratification of patients with pancreatic cancer and implied poor overall survival (Kim and Kim 2021; Toyama et al. 2020). However, Toyama et al. included patients with distant metastasis (Toyama et al. 2020), and these two studies did not perform PET harmonization (Kim and Kim 2021; Toyama et al. 2020). Toyama et al. also emphasized that GLZLM GLNU is a significant prognostic factor in the multivariate analysis for OS (Toyama et al. 2020), which is not consistent with our study. However, in our study, GLZLM LZLGE was a significant prognostic factor in both the first and second multivariate analyses for OS. GLZLM counts the number of groups (so-called zones) of interconnected neighbouring voxels with the same grey level (Ha et al. 2019; Mayerhoefer et al. 2020). For this reason, GLZLM GLNU increases if the tumour heterogeneity of pancreatic cancer increases (Zwanenburg et al. 2016). Then, GLZLM LZLGE is defined as the distribution of the long homogeneous zones with low grey levels (Ha et al. 2019), and this may be related to the tumour heterogeneity. Besides, GLCM contrast was borderline significant only in the first multivariate analyses for PFS in our study. GLCM captures spatial relationships of pair of voxels in different directions, and GLCM contrast emphasizes grey-level differences between voxels belonging to a voxel pair (Mayerhoefer et al. 2020), which can also be an indicator of tumour heterogeneity. Generally, pancreatic cancer can be classified into several subtypes, including classical, immunological, basal-like, and exocrine-like types (Martens et al. 2019). Basal-like pancreatic tumours, which are highly glycolytic, have acellular stroma, contain poorly differentiated tumours, and are associated with metastatic spread leading to poorer prognosis compared to classical tumours (Martens et al. 2019). Interestingly, heterogeneity exists even within the same patient with pancreatic cancer (Martens et al. 2019). Unfortunately, we could not demonstrate the prognostic significance of GLZLM GLNU, possibly because the number of patients in each batch was less than 20, which might have decreased the statistical power, as stated in previous publications (Goh et al. 2017; Nygaard et al. 2016; Orlhac et al. 2022; Da-Ano et al. 2020), as well as our small total sample size. For this reason, a multicentre study with larger sample sizes is warranted to investigate whether tumour heterogeneity of pancreatic cancer is correlated to poor prognosis.

In our study, MTV was the borderline significant and the significant prognostic factor in the first and the second multivariate analysis for PFS, respectively. Lee et al. demonstrated that MTV > 3.0 is a significant indicator of poor PFS (Lee et al. 2014), which is not consistent with our result. Of note, our cut-off of MTV determined with CART using R package was 20 in the patients with T2 and T3 stage. In our cohort, it means that even the patients with MTV ≥ 20 could undergo the curative surgery with no pathologically residual tumour regardless of their large tumour sizes, which should be taken into consideration in terms of tumour invasiveness.

In OS, neural invasion and Shape sphericity were significant in the first multivariate analysis, whereas Shape sphericity was borderline significant in the second multivariate analysis. Wei et al. also selected Shape sphericity as one of the optimal radiomic features to include into a formula of rad-score by using the least absolute shrinkage and selection operator (LASSO) algorithm with tenfold cross-validation (Wei et al. 2021). Shape sphericity describes how the tumour shape differs from a sphere (Mayerhoefer et al. 2020), which may be an indicator of tumour irregularity or invasiveness. Regarding neural invasion, Iwasaki et al. demonstrated that the patients of pancreatic cancer with high neural invasion showed statistically significantly shorter OS than the other patients (Iwasaki et al. 2019). These findings are consistent with our results.

Some studies investigated the clinical usefulness of FDG PET radiomic features of pancreatic cancer using machine learning (Wei et al. 2021; Toyama et al. 2020); however, Toyama et al. included patients with distant metastases (Toyama et al. 2020), and Wei et al. included patients with microscopically residual tumours after surgery (Wei et al. 2021). Furthermore, only a few FDG PET radiomic studies using harmonization techniques examined pancreatic cancer patients who underwent R0 curative surgery. Except for MTV and GLCM contrast for PFS, and Shape sphericity and GLZLM LZLGE for OS, our study could not identify significant prognostic indices among PET parameters. However, Buvat et al. emphasize the importance of publishing negative PET radiomic results of methodologically well-designed studies because these findings may be useful to avoid unnecessary and costly repetitions of already performed analyses (Buvat and Orlhac 2019). Thus, our results might facilitate future FDG PET radiomic prospective multicentre research on pancreatic cancer.

Adjuvant chemotherapy significantly improves PFS, as stated in a previous report (Uesaka et al. 2016). In particular, adjuvant chemotherapy with S-1 was reported to be more advantageous than gemcitabine considering prognosis and adverse effects (Uesaka et al. 2016). In PFS, age was the significant prognostic factor in the first multivariate analysis, which is difficult to explain. Interestingly in our study, however, there were 9 of 33 (27.3%) patients with the age > 70, and 14 of 25 (56.0%) patients with the age ≤ 70, who underwent the completion of adjuvant chemotherapy. This might be one of the contributors to this result. The completion of adjuvant chemotherapy is not a preoperative prognostic factor precisely, so we did not include it in our prognostic analyses for PFS and OS.

We did not generate a prognostic model as provided in other publications (Nakajo et al. 2021; Zhang et al. 2020) because of our concern regarding overfitting due to the small sample size (Ha et al. 2019). We just used random forest algorithm with the concept of Gini importance for the feature selection of the second multivariate analysis to determine useful prognostic factors using clinical information and PET radiomic features. Therefore, we did not perform a validation of the model in accordance with the TRIPOD statement (Moons et al. 2015), but our study was in accordance with the STROBE guidelines. When a prospective multicentre study with a larger sample size that enables prognostic model development becomes available, both internal and external validation for the prognostic model may be warranted (Nakajo et al. 2021; Moons et al. 2015).

As a limitation, the surgical date of the enrolled patients was between 2011 and 2020, possibly leading to variability in adjuvant chemotherapy protocols. The sample size was small because of the nature of our single-centre analysis.

## Conclusions

We investigated significant prognostic factors for PFS and OS, including harmonized PET radiomic features and clinical information, in patients with pancreatic cancer who underwent complete curative surgery. In addition to the age for PFS and neural invasion for OS, MTV and GLCM contrast for PFS, and Shape sphericity and GLZLM LZLGE for OS might be a prognostic indicator of the patients with pancreatic cancer who underwent curative surgery. A prospective multicentre analysis with a larger sample size may be warranted to confirm this result.

## Data Availability

The data sets generated and/or analysed during the current study are not publicly available due to regulations in our research protocol as approved by the institutional review board (IRB) in consideration of the patients’ privacy but may be available from the corresponding author upon reasonable request after permission by the IRB.

## Abbreviations

CA19-9: Cancer antigen 19-9
CEA: Carcinoembryonic antigen
CT: Computed tomography
EANM: European Association of Nuclear Medicine
FDG: Fluorodeoxyglucose
Gd-EOB-DTPA: Gadolinium-ethoxybenzyl diethylenetriamine pentaacetic acid
GLCM: Grey-level co-occurrence matrix
GLRLM: Grey-level run length matrix
GLZLM: Grey-level zone length matrix
MRI: Magnetic resonance imaging
MTV: Metabolic tumour volume
NGLDM: Neighbouring grey-level different matrix
PET: Positron emission tomography
PFS: Progression-free survival
OS: Overall survival
STROBE: Strengthening the report of observational studies in epidemiology
SUV: Standardized uptake value
S-1: TS-1
TLG: Total lesion glycolysis
TP: Total pancreatectomy
UICC: Union for International Cancer Control
